# The immunologic constant of rejection classification refines the prognostic value of conventional prognostic signatures in breast cancer

**DOI:** 10.1038/s41416-018-0309-1

**Published:** 2018-10-24

**Authors:** François Bertucci, Pascal Finetti, Ines Simeone, Wouter Hendrickx, Ena Wang, Francesco M. Marincola, Patrice Viens, Emilie Mamessier, Michele Ceccarelli, Daniel Birnbaum, Davide Bedognetti

**Affiliations:** 1 Equipe Oncologie Prédictive, Centre de Recherche en Cancérologie de Marseille (CRCM), Institut Paoli-Calmettes, INSERM UMR1068, CNRS UMR725 Marseille, France; 2 Département d’Oncologie Médicale, Institut Paoli-Calmettes, Marseille, France; 3 Faculté de Médecine, Aix-Marseille Université, Marseille, France; 4 Istituto Italiano di Tecnologia, Milan, Italy; 5 Research Branch, Sidra Medical and Research Center, Doha, Qatar; 6 Department of Science, University of Sannio, Benevento, Italy; 7 BIOGEM, Ariano Irpino, Italy; 8 Present Address: Abbvie Corporation, Redwood City, CA USA

## Abstract

**Background:**

The immunologic constant of rejection (ICR) is a broad phenomenon of Th-1 immunity-mediated, tissue-specific destruction.

**Methods:**

We tested the prognostic value of a 20-gene ICR expression signature in 8766 early breast cancers.

**Results:**

Thirty-three percent of tumours were ICR1, 29% ICR2, 23% ICR3, and 15% ICR4. In univariate analysis, ICR4 was associated with a 36% reduction in risk of metastatic relapse when compared with ICR1-3 (*p* = 2.30E–03). In multivariate analysis including notably the three major prognostic signatures (Recurrence score, 70-gene signature, ROR-P), ICR was the strongest predictive variable (*p* = 9.80E–04). ICR showed no prognostic value in the HR+/HER2− subtype, but prognostic value in the HER2+ and TN subtypes. Furthermore, in each molecular subtype and among the tumours defined as high risk by the three prognostic signatures, ICR4 patients had a 41–75% reduction in risk of relapse as compared with ICR1-3 patients. ICR added significant prognostic information to that provided by the clinico-genomic models in the overall population and in each molecular subtype. ICR4 was independently associated with achievement of pathological complete response to neoadjuvant chemotherapy (*p* = 2.97E–04).

**Conclusion:**

ICR signature adds prognostic information to that of current proliferation-based signatures, with which it could be integrated to improve patients’ stratification and guide adjuvant treatment.

## Background

Despite recent progresses, ~15% of patients with breast cancer still develop metastases and die. During the last decades, genomic analysis revealed the extent of the molecular heterogeneity of disease.^1^ Based on gene expression profiling, a new molecular classification was defined, confirming that breast cancer is a group of molecularly distinct subtypes associated with different clinical outcome and prognostic features. In parallel, multigene signatures prognostic and/or predictive for response to chemotherapy were developed.^2,3^ Several commercially available prognostic classifiers have been cleared by the Food and Drug Administration for clinical use or endorsed by American Society of Clinical Oncology (ASCO), National Comprehensive Cancer Network (NCCN), and Saint-Gallen guidelines to assist clinicians in making decisions about adjuvant chemotherapy, in particular for patients with HR+/HER2− tumour. Indeed, those signatures, mainly based on genes involved in cell proliferation, provide modest prognostic information for patients with classically proliferative HER2+ or triple-negative (TN) tumours.

The role of immunity in counteracting tumour progression is clearly recognised.^4,5^ Classically, breast cancer is considered less immunogenic than melanoma or renal cell carcinoma. Nonetheless, the role of immunity has emerged with the demonstration of a favourable predictive impact of the presence of tumour-infiltrating lymphocytes (TILs)^6^ and of gene expression signatures of immune response (IR), notably for TN and HER2+ tumours.^7,8^ Given the recent therapeutic success of immune checkpoint inhibitors in several types of cancers,^9,10^ these drugs were tested in breast cancer:^11^ no or very low activity was observed in HR+ tumours, whereas higher activity was reported in small subsets of heavily pre-treated TN tumours preselected with an increased PD-L1 expression with respective 18.5 and 24% objective response rates with pembrolizumab (*n* = 27)^12^ and atezolizumab (*n* = 21),^13^ and remarkably durable responses.

Recent data suggest that not only the composition of tumour-infiltrating immune cells, but also their functional orientation might serve as a prognostic/predictive marker to select systemic therapies.^5^ The functional orientation towards cytotoxic response is observed in tumours undergoing regression following immunotherapy and, in melanoma, has been associated with responsiveness to interleukin-2, adoptive therapy, vaccines, and checkpoint inhibitors.^14–19^ Although prognostic immune signatures defined in breast cancer differ in term of gene composition, most of them include transcripts underlying a cytotoxic response.^20–22^ The corresponding pathways are also activated during other forms of immunity-mediated tissue-specific destruction, such as allograft rejection,^23^ graft-versus-host disease,^24^ and flares of autoimmunity.^25^ We defined them as the immunologic constant of rejection (ICR).^5,18^ More specifically, the ICR consists in a signature including genes involved in Th-1 signalling interferon (*IFNG*, *TBX21*, *CD8A/B*, *IL12B*, *STAT1*, and *IRF1*), Th-1 chemoattraction (such as the CXCR3 and CCR5 ligands, respectively, *CXCL9* and *CXCL10*, and *CCL5*), and cytotoxic functions (*GNLY*, *PRF*, *GZMA*, *GZMB*, and *GZMH*). Interestingly, the expression of these pro-cytotoxic transcripts in tumours is associated with the counter activation of suppressive mechanisms, such as the expression of *IDO1*, *CTLA4*, *CD274 (PD-L1)*, *PDCD1 (PD-1)*, and *FOXP3.*^26^ In a study^27^ centred on the TCGA data set, we found that breast cancers can be classified in four classes according to the ICR signature. In such classification, the level of immune antitumour response progressively decreased from ICR4 to ICR1. The ICR4 tumours, characterised by the coordinate activation of the ICR pathways, displayed a prolonged survival as compared with ICR1-3 tumours in univariate analysis.

Here, to further asses its clinico-biological value, we expanded the ICR classification to a set of 8766 non-metastatic, invasive primary breast cancers. We searched for correlations with clinico-biological data, including metastasis-free survival (MFS) and pathological complete response (pCR) to neoadjuvant chemotherapy.

## Materials and methods

### Breast cancer samples and gene expression profiling

Our institutional series included 352 tumour samples from pre-treatment invasive primary mammary carcinomas either surgically removed or biopsied.^28^ The study was approved by our institutional review board. Each patient had given a written informed consent for research use. Samples had been profiled using Affymetrix U133 Plus 2.0 human microarrays (Santa Clara, CA, USA). We pooled them with 34 public breast cancer data sets comprising both gene expression profiles generated using DNA microarrays and RNA-Seq and clinicopathological annotations. These sets were collected from the National Center for Biotechnology Information (NCBI)/Genbank GEO and ArrayExpress databases, and authors’ website (Supplementary Table 1). The final pooled data set included 8766 non-redundant non-metastatic, non-inflammatory, primary, invasive breast cancers.

### **Gene expression data analysis**

Before analysis, several steps of data processing were applied. The first step was the normalisation of each set separately. It was done in R using Bioconductor and associated packages; we used quantile normalisation for the available processed data from non-Affymetrix-based sets (Agilent, SweGene, and Illumina), and Robust Multichip Average (RMA) with the non-parametric quantile algorithm for the raw data from the Affymetrix-based sets. In the second step, we mapped the hybridisation probes across the different technological platforms represented as previously reported.^29^ When multiple probes mapped to the same GeneID, we retained the most variant probe in a particular data set. We log2 transformed the available TCGA RNA-Seq data that were already normalised.

In order to avoid biases related to trans-institutional immunohistochemistry analyses and thanks to the bimodal distribution of respective mRNA expression levels, the Estrogen Receptor (ER), progesterone receptor (PR), and HER2 statutes (negative/positive) were defined on transcriptional data of *ESR1, PGR*, and *HER2*, respectively, as previously described.^30^ The molecular subtypes of tumours were defined as HR+/HER2− for ER+ and/or PR+ and HER2− tumours, HER2+ for HER2+ tumours, and TN for ER−, PR−, and HER2− tumours.

We applied in each data set separately several multigene signatures. First, the ICR classifier based on consensus clustering (CC) analysis of the expression levels of 20 representative immune genes (namely, *CCL5, CD274, CD8A, CD8B, CTLA4, CXCL9, CXCL10, FOXP3*, *GNLY, GZMA, GZMB, GZMH, IDO1, IFNG, IL12B, IRF1, PDCD1, PRF1, STAT1*, and *TBX21*) as previously described.^27^ Briefly, the CC analysis was performed in R using the Bioconductor package “ConsensusClusterPlus”^31^ setting as input parameters 5000 repetitions, 80% item resampling (pItem), a number of groups (k) fixed to 4 (in order to have all data sets stratified with the same number of classes, 4 being the optimal number of groups for the TCGA cohort,^27^) and the use of an agglomerative hierarchical clustering with ward criterion (Ward.D2) inner and complete outer linkage. We also applied the three major prognostic multigene classifiers of breast cancer: Recurrence score,^32^ 70-gene signature,^33^ and Risk of Relapse score based on PAM50 subtype and proliferation Risk of Relapse (ROR-P).^2^ Other signatures included the metagenes associated with immune cell populations such as T cells, CD8+ T cells and B cells defined by Palmer et al.,^34^ the transcriptional signatures of 24 different innate and adaptative immune cell subpopulations defined by Bindea et al.,^35^ the cytolytic activity score,^36^ the activation score of IFNα, IFNγ, and tumor necrosis factor (TNFα) immune-related and TP53 biological pathways,^37^ and a chromosomal instability signature.^38^ We also applied to each data set separately three immune gene signatures reported as prognostic in specific molecular subtypes of breast cancer: the IR signature^22^ and the lymphocyte-specific kinase (LCK) signature^20^ in ER− breast cancers, the Immune 28-kinase signature^21^ in basal/TN breast cancers, and the LCK signature^20^ in HER2+ breast cancers. Finally, we calculated the mitogen-activated protein kinase (MAPK)-mut score using MAPK genes upregulated in MAPK2K4 /MAP3K1 mutated vs. wild-type tumours, as listed elsewhere.^27^

### Statistical analysis

Correlations between tumour classes and clinicopathological variables were analysed using the one-way analysis of variance (ANOVA) or the Fisher’s exact test when appropriate. MFS was calculated from the date of diagnosis until the date of distant relapse. Follow-up was measured from the date of diagnosis to the date of last news for event-free patients. Survivals were calculated using the Kaplan–Meier method and curves were compared with the log-rank test. Uni- and multivariate prognostic analyses for MFS were done using Cox regression analysis (Wald test). The variables submitted to univariate analyses included patients’ age at diagnosis ( ≤ 50 years vs. > 50), pathological type (lobular vs. ductal vs. other), pathological axillary lymph node status (pN: negative vs. positive), pathological tumour size (pT1 vs. pT2 vs. pT3), pathological grade (1 vs. 2 vs. 3), molecular subtypes (HR+/HER2− vs. HER2+ vs. TN), and classifications based on ICR and prognostic multigene signatures. The likelihood ratio (LR) tests were used to assess the prognostic information provided beyond that of a clinical model and other signatures, assuming a *χ*^2^ distribution. Changes in the LR values (LR-ΔX^2^) measured quantitatively the relative amount of information of one model compared with another. We also analysed the pCR after neoadjuvant chemotherapy, defined as absence of invasive cancer in both breast and axillary lymph nodes. Uni- and multivariate analyses for pCR were done using logistic regression. Variables with a *p*-value < 0.05 in univariate analyses were tested in multivariate analyses. All statistical tests were two sided at the 5% level of significance. Statistical analysis was done using the survival package (version 2.30) in the R software (version 2.9.1; http://www.cran.r-project.org/). We followed the reporting REcommendations for tumour MARKer prognostic studies (REMARK criteria).^39^

## Results

### Breast cancer population and ICR classification

We applied the ICR classification to a series of 8766 pre-treatment cancer samples. Most of the patients were >50 years old and most of the tumours were ductal type, pT1–pT2, pN−, grade 2–3, ER+, HER2− (Supplementary Table 2**)**. Sixty-six percent were HR+/HER2−, 12% were HER2+, and 22% were TN. ICR classification defined 2874 tumours (33%) as ICR1, 2516 (29%) as ICR2, 2061 (23%) as ICR3, and 1315 (15%) as ICR4, with progressive decrease of the enrichment of the ICR signature from ICR4 to ICR1. The box plot of expression of each ICR gene according to the ICR classes is shown in Supplementary Figure 1.

### ICR classification and clinicopathological and biological features

We found correlations between the ICR classes and all tested clinicopathological features (Supplementary Table 3). ICR4 class was associated with age ≤ 50 years, ductal type, less pT1, less pN0, high grade, ER− status, PR− status, and TN subtypes. Interestingly, for all those correlations, a continuum existed from ICR1 to ICR4. The TN subtype was more enriched in ICR4 (28%) than the HER2+ subtype (19%), which was also more enriched than the HR+/HER2− subtype (10%; *p* < 1.00E–06).

**Fig. 1:**
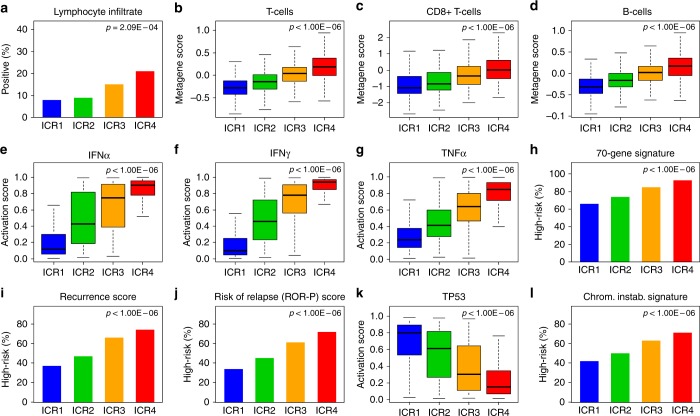
Correlations of ICR classes with immunity-related parameters and prognostic gene expression signatures of breast cancer. For each ICR class, are indicated the percentage of samples with high lymphocyte infiltrate score samples (**a**), metagene expression scores of T cells (**b**), CD8+ cells (**c**), and B cells (**d**), activation score of IFNα (**e**), IFNγ (**f**), and TNFα (**g**) pathways, percentage of high-risk samples according to the 70-gene signature (**h**), the Recurrence score (**i**), and the Risk of Relapse (ROR-P) score (**j**), activation score of P53 pathway (**k**), and percentage of high-risk samples according to the chromosomal instability signature (**l**). The *p*-values are indicated (Fisher’s exact test or ANOVA test when appropriate)

Correlations also existed with immunity-related factors and prognostic signatures of breast cancer (Supplementary Table 4). We found a positive correlation with the lymphocyte infiltrate scored binary (low vs. high), the percentage of high-score samples increasing with the ICR class (*p* = 2.09E–04). We found strong positive correlation (*p* < 1.00E–06) with immune gene expression signatures defined in breast cancer: the metagene scores of T cells, CD8+ T cells, and B cells^34^ increased from ICR1 to ICR4, as did the activation score of IFNα, IFNγ, and TNFα pathways^37^ (Fig. 1), and the cytolytic activity score.^36^ This immune pattern was confirmed and refined using the 24 Bindea signatures for immune cell subsets,^35^ showing a strong enrichment from ICR1 to ICR4 for T cells, cytotoxic T cells, CD8+ T cells, T-helper cells, and Tγδ cells, activated NK CD56^dim^ cells and neutrophils (*p* < 1.00E–100; Supplementary Figure 2). Among T-helper cells, the Th-1/Th-2 ratio increased from ICR1 to ICR4, whereas Th-17 enrichment, often associated with unfavourable prognosis,^35,40^ decreased. This antitumour activation was also correlated to subsets involved in antigen presentation, such as activated dendritic cells (aDCs), DC, B cells, and macrophages. Mast cells and eosinophils decreased from ICR1 to ICR4. Finally, the percentage of high-risk samples increased from ICR1 to ICR4 (*p* < 1.00E–06) for the 70-gene signature,^33^ the Recurrence score,^32^ and the ROR-P score^2^ (Fig. 1).

The activation score of TP53 pathway^37^ decreased from ICR1 to ICR4, whereas the percentage of samples with chromosomal instability, as defined by the Carter signature,^38^ increased (Fig. 1). The MAPK-mut score, reflection of the degree of MAPK deregulation, decreased from ICR1 to ICR4 in the whole series and in each molecular subtype separately (Supplementary Figure 3), as previously reported.^27^ Here too, for all tested signatures, a continuum was present between the four classes. Of note, all correlations with the ICR classes remained significant after adjustment on the molecular subtype (Supplementary Table 5).

### ICR classification **and MFS in the whole population**

**Fig. 2:**
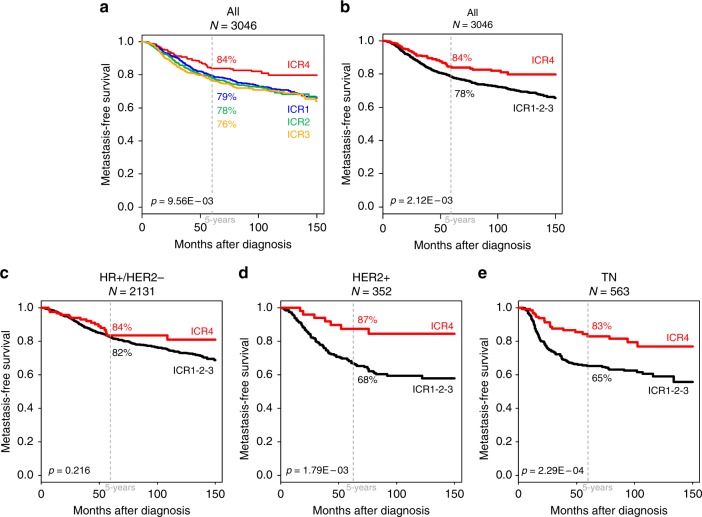
Metastasis-free survival according to the ICR classification in breast cancer. **a** Kaplan–Meier MFS curves in all patients according to the four ICR classes. **b** Similar to (**a**), but after pooling the ICR1, 2 and 3 classes in the ICR1-3 class. **c-e** Similar to (**b**), but in the HR+/HER2− subtype (**c**), the HER2+ subtype (**d**, and the TN subtype (**e**). The *p*-values are indicated (log-rank test)

We assessed the prognostic value of the ICR classification in term of MFS in the 3046 informative patients non-metastatic at diagnosis, operated and without neoadjuvant systemic therapy: 2415 remained metastasis-free during a median follow-up of 72 months (range, 1–299) and 631 displayed metastatic relapse. The 5-year MFS rate was 79% (95% CI, 77–81). As shown in Fig. 2a, the MFS was different among the four classes: 79% (95% CI, 77–82) in ICR1, 78% (95% CI, 75–81) in ICR2, 76% (95% CI, 73–80) in ICR3, and 84% (95% CI, 80–89) in ICR4 (*p* = 9.56E–03, log-rank test). Based on the absence of difference in MFS between the ICR1, 2, and 3 classes (*p* = 0.39, log-rank test), we pooled them into the ICR1-3 class.

In univariate analysis (Table 1), the ICR classification was associated with MFS: patients in the ICR4 class showed longer 5-year MFS (84% [95% CI, 80–89]) than patients in the ICR1-3 class (78%; [95% CI, 76–80]; *p* = 2.12E–03, log-rank test; Fig. 2b), representing a 36% reduction in the risk of relapse (hazard ratio (HR) = 0.64, 95% CI, 0.47–0.85, *p* = 2.30E–03, Wald test). Variables associated with shorter MFS included higher pathological size, higher grade, and TN and HER2+ subtypes. In multivariate analysis, all these variables remained significant, including the ICR classification (*p* = 4.76E–03, Wald test). Importantly, the prognostic value of the ICR score also persisted in the multivariate analysis (HR = 0.53, 95% CI, 0.36–0.77, *p* = 9.80E–04, Wald test; Table 1), which included the three major prognostic signatures of breast cancer (70-gene signature, Recurrence score, ROR-P), suggesting independent prognostic value of ICR classification compared with these signatures. Of note (data not shown), the ICR classification was associated with both early (0–5 years) metastatic relapses (HR = 0.67, 95% CI, 0.49–0.91, *p* = 1.16E–02, Wald test) and late ( > 5 years) metastatic relapses (HR = 0.43, 95% CI, 0.20–0.91, *p* = 2.85E–02, Wald test).

**Table 1:** Univariate and multivariate Cox regression analyses for MFS in breast cancer

		Univariate			Multivariate		
Characteristics		*N*	Hazard ratio [95% CI]	*p*-Value	*N*	Hazard ratio [95% CI]	*p*-Value
Age (years)	>50 vs. ≤50	2265	0.89 [0.74–1.08]	0.250			
Pathological type	Lobular vs. ductal	1530	0.83 [0.52–1.33]	0.892			
	Mixed vs. ductal		0.97 [0.48–1.97]				
	Other vs. ductal		0.93 [0.54–1.61]				
Pathological tumour size (pT)	pT2 vs. pT1	2262	1.47 [1.19–1.81]	0.001	1228	1.29 [1.02–1.63]	0.030
	pT3 vs. pT1		1.57 [1.07–2.31]		1228	1.74 [1.05–2.86]	0.030
Pathological axillary node status (pN)	Positive vs. negative	2765	1.18 [1–1.4]	0.053			
Pathological grade	2 vs. 1	1566	2.04 [1.43–2.91]	< 1.00E–06	1228	1.42 [0.96–2.11]	0.081
	3 vs. 1		3.98 [2.83–5.60]		1228	1.97 [1.31–2.97]	0.001
Molecular subtype	HER2+ vs. HR+/HER2−	3046	1.64 [1.32–2.05]	0.000	1228	1.07 [0.75–1.52]	0.717
	TN vs. HR+/HER2−		1.74 [1.43–2.10]		1228	0.92 [0.66–1.28]	0.607
70-gene signature, risk	Poor vs. good	3046	2.45 [1.95–3.08]	0.000	1228	1.06 [0.72–1.55]	0.778
Recurrence score, risk	Poor vs. good	3046	2.21 [1.83–2.67]	0.000	1228	1.68 [1.15–2.46]	0.007
	Intermediate vs. good		1.78 [1.41–2.25]		1228	1.79 [1.27–2.54]	0.001
Risk of relapse, ROR-P	Poor vs. good	3046	2.58 [2.12–3.14]	< 1.00E–06	1228	1.69 [1.15–2.48]	0.007
	Intermediate vs. good		1.94 [1.50–2.52]		1228	1.44 [0.96–2.15]	0.075
ICR classification	ICR4 vs. ICR1-3	3046	0.64 [0.47–0.85]	0.002	1228	0.53 [0.36–0.77]	0.001

### ICR classification and MFS in each molecular subtype

In order to further assess the complementarity of ICR classification with other signatures, we repeated the same analysis in each molecular subtype separately (Supplementary Table 6, Figs. 2c-e). In the HER2+ subtype (*n* = 352), the ICR classification and the LCK signature were associated with MFS in univariate analysis, with a HR for MFS event equal to 0.31 (95% CI, 0.15–0.68; *p* = 3.14E–03, Wald test) in ICR4 when compared with ICR1-3. In multivariate analysis, only the ICR classification remained significant. In the TN subtype (*n* = 563), ICR classification displayed strong prognostic value with a HR for MFS event equal to 0.44 (95% CI, 0.28–0.69; *p* = 3.42E–04, Wald test) in ICR4 when compared with ICR1-3. The other immune signatures (IR, LCK, and 28-kinase) were also significant in univariate analysis, but in multivariate analysis, only the ICR signature kept its prognostic value (*p* = 1.57E–02, Wald test). Finally, in the HR+/HER2− subtype (*n* = 2131), the ICR classification was not associated with MFS, whereas most of the clinicopathological variables and all classical prognostic signatures were.

**Fig. 3:**
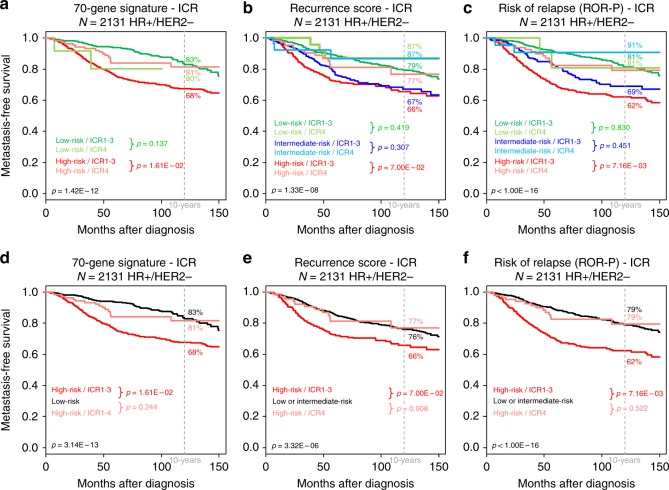
Metastasis-free survival according to the ICR classification within the different risk groups defined by three proliferation-based prognostic signatures in the 2131 HR+/HER2− patients. Kaplan–Meier MFS curves in patients according to the ICR1-3 and the ICR4 classes in the HR+/HER2− subtype and according to the relapse risk groups defined by the 70-gene signature (**a**), the Recurrence score (**b**), and the ROR-P score (**c**). **d– f** Similar to (**a**–**c**), respectively, but the low-risk and intermediate-risk curves are not stratified according to ICR and are pooled for (**e**) and (**f**). The *p*-values are indicated (log-rank test)

These three commercial gene signatures are mainly used to decide whether HR+/HER2− patients need adjuvant chemotherapy (high risk) or not (low risk). We investigated whether the ICR signature could identify prognostic classes within the different risk groups defined by those signatures in the 2131 HR+/HER2− patients. A prognostic value existed in the high-risk groups, but not in the intermediate-risk groups, and even less in the low-risk groups (Fig. 3). In the high-risk group defined by the 70-gene signature (*n* = 1414), the HR for MFS event was 0.56 (95% CI, 0.35–0.90; *p* = 1.76E–02, Wald test) in ICR4 (9% of samples) when compared with ICR1-3. In the Recurrence score-defined high-risk group (*n* = 585), the corresponding HR was 0.59 (95% CI, 0.33–1.05; *p* = 7.47E–02, Wald test) with 13.5% of samples in ICR4, and in the ROR-P score-defined high-risk group (*n* = 871), it was 0.50 (95% CI, 0.30–0.84; *p* = 8.33E–03, Wald test) with 10.5% of samples in ICR4. Interestingly, for each signature, the MFS curves were not significantly different between the high-risk/ICR4 class and the low-risk or low/intermediate-risk group, but significantly differed from those of the high-risk/ICR1-3 class. For example regarding the Recurrence score, the 10-year MFS were 76% (95% CI, 73–79) in the low/intermediate-risk group, 77% (95% CI, 65–72) in the high-risk/ICR4 class, and 66% (95% CI, 60–72) in the high-risk/ICR1-3 class. For the ROR-P score, the corresponding 10-year MFS were 79% (95% CI, 76–82), 79% (95% CI, 70–90), and 62% (95% CI, 58–67).

The same analysis gave similar results in the HER2+ and TN subtypes. In these subtypes, the low-risk and intermediate-risk groups were the minority and were pooled for analysis. A prognostic value existed in the high-risk groups, but not in the low or low/intermediate-risk groups. In the 352 HER2+ patients, for the 70-gene signature, the 10-year MFS were 85% (95% CI, 75–96) in the high-risk/ICR4 class, and 61% (95% CI, 54–68) in the high-risk/ICR1-3 class. For the Recurrence score, the 10-year MFS were 87% (95% CI, 77–98) in the high-risk/ICR4 class, and 58% (95% CI, 51–66) in the high-risk/ICR1-3 class. For the ROR-P score, the corresponding 10-year MFS were 86% (95% CI, 76–98) and 59% (95% CI, 51–67; Supplementary Figure 4). Regarding the 563 TN patients, for the 70-gene signature, the 10-year MFS were 77% (95% CI, 68–87) in the high-risk/ICR4 class, and 59% (95% CI, 53–66) in the high-risk/ICR1-3 class. For the Recurrence score, the 10-year MFS were 77% (95% CI, 68–87) in the high-risk/ICR4 class, and 59% (95% CI, 53–66) in the high-risk/ICR1-3 class. For the ROR-P score, the corresponding 10-year MFS were 78% (95% CI, 69–88), and 65% (95% CI, 53–80).

### Comparison of ICR classification with other prognostic signatures

Such prognostic complementarity between the proliferation-based signatures and our ICR signature was tested using the LR tests (Table 2). In the overall population and in each molecular subtype, significant additional prognostic information was provided by our signature beyond that provided by the clinical model combined to each other signature (70-gene, Recurrence score, and ROR-P score). For example, ICR signature added information to that provided by the combination of clinical model and Recurrence score in the overall population (LR-ΔX^2^ = 10.39, *p* = 1.27E–03), in the TN (LR-ΔX^2^ = 15.68, *p* = 7.52E–05), HER2+ (LR-ΔX^2^ = 12.72, *p* = 3.63E–04), and HR+/HER2− subtypes (LR-ΔX^2^ = 4.46, *p* = 3.46E–02). Based on the LR-ΔX^2^ values, the added prognostic information was larger in the TN and HER2+ subtypes and the overall population than in the HR+/HER2− subtype where, however, it was significant.

**Table 2:** Comparison of prognostic information for MFS

Patient group		All	TN	HER2+	HR+/HER2−
N° patients		3046	563	352	2131
N° distant Evt		631	143	97	391
Clinical model	LR-X²	75.00	---	---	45.45
	*p*-Value	3.84E–14	---	---	3.21E–09
ICR classification	LR-X²	10.57	15.38	12.19	1.66
	*p*-Value	1.15E–03	8.79E–05	4.81E–04	0.198
70-gene signature	LR-X²	72.09	0.11	1.08	55.09
	*p*-Value	< 2.00E–16	0.735	0.298	1.15E–13
Clinical + 70-gene + ICR4	LR-X²	90.2	15.41	12.66	61.01
	*p*-Value	< 2.00E–16	4.51E–04	1.78E-03	2.81E–11
Clinical + 70-gene + ICR4 vs. Clinical + 70-gene	LR-ΔX²	10.31	15.30	11.57	3.78
	*p*-Value	1.33E–03	9.19E–05	6.69E–04	0.052
Recurrence score (RS)	LR-X²	73.76	0.53	2.35	38.44
	*p*-Value	1.11E–16	0.769	0.309	4.49E–09
Clinical + RS + ICR4	LR-X²	110.90	16.20	15.06	75.67
	*p*-Value	< 2.00E–16	1.03E–03	1.76E–03	1.05E–13
Clinical + RS + ICR4 vs. Clinical + RS	LR-ΔX²	10.39	15.68	12.72	4.46
	*p*-Value	1.27E–03	7.52E–05	3.63E–04	3.46E–02
Risk of relapse (ROR-P)	LR-X²	103.20	0.65	3.02	77.38
	*p-*Value	< 2.00E–16	0.723	0.221	< 2.00E–16
Clinical + ROR-P + ICR4	LR-X²	106.5	16.12	16.11	74.18
	*p*-Value	< 2.00E–16	1.07E–03	1.08E–03	2.10E–13
Clinical + ROR-P + ICR4 vs. Clinical + ROR-P	LR-ΔX²	12.41	15.47	13.09	5.77
	*p*-Value	4.27E–04	8.38E–05	2.97E–04	1.63E–02
* Clinical model variables		*pT, grade & HR mRNA*	*none*	*none*	*pT & grade*

*the clinical variables mentioned correspond to the variables significant in multivariate analysis and integrated in the Clinical model.

### ICR classification and pathological response to chemotherapy

A total of 1229 breast cancer samples were informative regarding the pathological response to anthracycline-based neoadjuvant chemotherapy. Among them, 283 (23%) displayed pCR, whereas 946 did not. In univariate analysis (Table 3), ICR classification was associated with pCR (43% pCR in ICR4 class vs 20% in the ICR1-3 class, *p* = 2.88E–10), with an odds ratio (OR) for pCR equal to 2.99 (85%CI 2.24–3.97). The other significant variables were high grade, and HER2+ and TN subtypes. In multivariate analysis, all variables remained significant, including the ICR classification (*p* = 2.97E–04, logit function). Here too, a continuum existed in term of pCR rate between the four ICR classes, from 14% (ICR1) to 20% (ICR2), 28% (ICR3), and 43% (ICR4). Such correlation between ICR classes and pCR rate was maintained in each molecular subtype separately (Supplementary Table 7).

**Table 3:** Univariate and multivariate analyses for pathological complete response to neoadjuvant chemotherapy in breast cancer

Characteristics		Univariate			Multivariate		
		*N*	Odds ratio [95% CI]	*p*-Value	*N*	Odds ratio [95% CI]	*p*-Value
Age (years)	>50 vs. ≤50	1227	0.84 [0.67–1.05]	0.210			
Pathological type	Lobular vs. ductal	526	1.69 [0.62–4.31]	0.368			
	Mixed vs. ductal	526	0.74 [0.36–1.42]	0.476			
	Other vs. ductal	526	0.82 [0.44–1.46]	0.593			
Pathological grade	2 vs. 1	1118	3.27 [1.15–14.3]	0.108	1118	2.42 [0.84–10.6]	0.235
	3 vs. 1	1118	11.5 [4.16–49.5]	7.58E–04	1118	5.86 [2.07–25.5]	1.61E–02
Molecular subtype	HER2+ vs. HR+/HER2−	1229	3.98 [2.83–5.60]	2.46E–11	1118	3.12 [2.14–4.54]	6.09E–07
	TN vs. HR+/HER2−	1229	3.50 [2.70–4.57]	5.76E–15	1118	2.56 [1.90–3.47]	2.99E–07
ICR classification	ICR4 vs. ICR1-3	1229	2.99 [2.24–3.97]	2.88E–10	1118	1.99 [1.45–2.72]	2.97E–04

Based on these results and the MFS results, we postulated that the prognostic value of ICR classification could be mediated, at least in part, by its association with response to chemotherapy. Thus, we analysed its prognostic value in our MFS data set according to the delivery or not of adjuvant chemotherapy, which was informed for 2355 patients, including 1653 HR+/HER2−, 265 HER2+, and 437 TN. As shown in Supplementary Figure 5, in the whole population the prognostic value was present in the chemotherapy-treated group (*p* = 1.40E–02, log-rank test), but not in the chemotherapy-naive group (*p* = 0.18); however, interaction was not significant (*p* = 0.14). Analysis per molecular subtype revealed no prognostic value for ICR classification in both groups in the HR+/HER2− patients and no interaction; by contrast, interaction was significant (*p* = 4.71E–02) in the TN patients, with strong prognostic value in the chemotherapy-treated group (*p* = 1.80E–03, log-rank test) and no prognostic value in the chemotherapy-naive group (*p* = 0.47); in HER2+ patients, there was no significant interaction, with strong prognostic value in the chemotherapy-naive group (*p* = 2.84E–02, log-rank test) and no prognostic value in the chemotherapy-treated group despite strong difference in MFS between the two ICR classes (*p* = 0.21). Thus, these data confirm that, in breast cancer, the ICR4 class is associated with higher response to chemotherapy, particularly in the TN subtype.

## Discussion

Here, we show that the transcriptional ICR signature, reflecting an immune antitumour response, defines a continuum of clinically and biologically relevant classes of breast cancers. The signature is associated with classical prognostic features and immunity-related parameters, and with MFS, where it refines the prognostic value of classical prognostic signatures, and with pathological response to chemotherapy.

Our approach tested the prognostic and predictive value for our signature in an independent series of samples, thus avoiding the problem of overfitting. We analysed a retrospective pooled set of 8766 pre-therapeutic samples of non-metastatic and invasive primary breast cancers, including 3046 cases informative for MFS and 1229 for pathological response to chemotherapy. Such figures allowed testing our hypothesis in uni- and multivariate analyses in the whole population, but also in each molecular subtype separately. Moreover, the whole-genome transcriptional data allowed testing several other gene signatures and modules relevant to breast cancer.

An immunological continuum was observed with increasing enrichment, from ICR1 to ICR4, of scores reflecting the presence of an antitumour IR, such as lymphocyte infiltrate, expression signatures of immune cell types including T cells, cytotoxic T cells, Th-1 cells, CD8+ T cells, T-helper cells, Tγδ cells, and antigen-presenting cells, and scores of IFNγ pathway activation and of cytolytic activity. Although the molecular subtype is classically associated with immunologic infiltrate, such correlations persisted in multivariate analysis including the molecular subtypes. The level of immune activation captured by the ICR classification positively correlated with classical negative prognostic features of breast cancer, as the scores of standard prognostic signatures (70-gene signature, Recurrence score, and ROR-P score). Here too, a continuum was observed from ICR1 to ICR4, the latter being associated with the poorer-prognosis features. The activation score of TP53 pathway^37^ decreased from ICR1 to ICR4, in agreement with the higher rate of inactivating *TP53* mutations reported in ICR4,^27^ whereas chromosomal instability^38^ increased.

Importantly, although associated with poor-prognosis features (including the TN subtype and high-risk defined by classical prognostic signatures), the ICR4 class displayed longer MFS than the three other classes, which showed similar MFS and were pooled. In the whole population, the 5-year MFS was 84% in ICR4 and 78% in pooled ICR1-3, with a HR for relapse equal to 0.64. Multivariate analysis showed that such prognostic value was independent from that of classical prognostic variables and of the three major prognostic signatures of breast cancer, clearly suggesting that IR (reflected by our classification) and tumour cell proliferation (reflected by the three other signatures) provide complementary prognostic information. Of note, the lymphocyte infiltration, relatively simple measure of IR, which was available only for the 999 TCGA samples, including 929 with available follow-up (88 HER2+, 180 TN, and 661 HR+/HER2−), was not associated with MFS in univariate analysis, whereas our ICR classification was (data not shown). In fact, the prognostic value of our ICR classification was dependent upon the molecular subtype of samples and complementary to that of other signatures: it was present in the TN and HER2+ subtypes, classically highly proliferative, but absent in the HR+/HER2− subtype, classically less proliferative. The opposite was observed for the three proliferation-based signatures currently used in clinical practice in patients with HR+/HER2− disease. Nevertheless, in HR+/HER2− patients, ICR stratified into prognostic classes the high-risk patients defined by these three signatures. Between 9 and 14% of high-risk patients (according to the signatures tested) were classified ICR4, and such patients had a 41–50% reduction of risk of distant relapse as compared with ICR1-3 high-risk patients. Interestingly, high-risk ICR4 patients exhibited high 10-year MFS (between 77 and 81%), similar to the 10-year MFS of low-risk or low/intermediate-risk patients. Regarding the TN and HER2+ subtypes, no prognostic signature is marketed to date. However, we included in our prognostic analysis three immune signatures centred on the antitumour response and previously reported as prognostic (IR, LCK, 28-kinase): we confirmed their prognostic value in univariate analysis, which was, however, lost in multivariate analysis when confronted to our ICR classification. Interestingly, in these subtypes also, ICR stratified into prognostic classes the high-risk patients defined by the three proliferation-based commercial signatures. In the HER2+ subtype, 17–18% of high-risk patients (according to the signatures tested) were classified ICR4, and such patients had a 68–75% reduction of risk of distant relapse as compared with ICR1-3 high-risk patients. Similarly in the TN subtype, 27–30% of high-risk patients (according to the signatures tested) were classified ICR4, with a 56–60% reduction of risk of distant relapse as compared with ICR1-3 high-risk patients. Clearly, our ICR signature added substantial prognostic information beyond that provided by the combination of clinical model and each major prognostic multigene signature.

Finally, the ICR classification was also independently associated with pathological response to anthracycline-based chemotherapy, with 43% pCR rate in ICR4 vs*.* 20% in ICR1-3, and an OR close to 3. Here too, there was a continuum between ICR1 and ICR4 in term of pCR rate, further linking the degree of antitumour response to the degree of chemosensitivity of breast cancer.^41,42^ Such correlation was observed in each molecular subtype. Unfortunately, no expression data are currently available in the literature for testing the eventual value of our signature as predictor for response to checkpoint inhibitors.

In conclusion, our 20-gene ICR signature displays robust predictive values for MFS and for pathological response to anthracycline-based chemotherapy in breast cancer. Among aggressive tumours, those with a coordinated antitumour response (ICR4) display better prognosis and better respond to chemotherapy than those without, further reinforcing the fact that immune reaction is an important component of breast cancer and complementary to cell proliferation in prognostic term. Our study displays several strengths: (i) the large size of the series, which represents to our knowledge one of the largest series reported so far analysing the prognostic/predictive value of gene signatures in breast cancer; (ii) the analysis per molecular subtype, demonstrating that the prognostic value is absent in the whole population of HR+/HER2− tumours, but major in the TN tumours; (iii) the persistence of prognostic and predictive values in multivariate analysis including classical prognostic signatures; (iv) the analysis per relapse risk in each molecular subtype, demonstrating that the prognostic value is present in high-risk tumours only; (v) the added prognostic value beyond that provided by the clinical model and each major prognostic signature; (vi) the biological relevance of the signature, which reveals a gradient of antitumour IR in breast cancer and suggests the potential therapeutic interest of stimulating a pro-Th-1 response; (vii) the small number of genes in the signature, which should facilitate its clinical application by using other tests applicable to formaldehyde-fixed paraffin-embedded samples such as quantitative reverse transcriptase-PCR. The main limitation is the retrospective nature of our series and associated biases.

The perspectives are therapeutic. Indirectly, the integration of ICR classification with classical prognostic signatures can improve prognostication of breast cancer. For example, identification of poor or good-prognosis cases within operated TN breast cancers should help tailor the systemic treatment: although the 5-year MFS of ICR4 class remains insufficient (83%) and cannot preclude the use of adjuvant chemotherapy, the strong MFS difference suggests that the ICR1-3 patients should need a more aggressive treatment than ICR4 patients. The same is true in HR+/HER2− patients defined as high risk according to the classical prognostic signatures. Such hypothesis should be tested prospectively to identify additional women that might be spared from unnecessary chemotherapy, or perhaps, which can be treated with adjuvant immune-modulatory approaches. More directly, since the antitumour IR seems to play a pivotal role regarding the clinical outcome, the manipulation of genes and/or pathways^11,43^ interfering with its development should provide new therapeutic weapons for treating these poor-prognosis tumours.

## Electronic supplementary material

Supplementary Figure 1

Supplementary Figure 2

Supplementary Figure 3

Supplementary Figure 4

Supplementary Figure 5

Supplementary Table 1

Supplementary Table 2

Supplementary Table 3

Supplementary Table 4

Supplementary Table 5

Supplementary Table 6

Supplementary Table 7
