# Supplementary material for: The immunologic constant of rejection classification refines the prognostic value of conventional prognostic signatures in breast cancer
Source: Br J Cancer. 2018 Oct 24;119(11):1383–91. doi: 10.1038/s41416-018-0309-1 (PMC6265245; doi:10.1038/s41416-018-0309-1)
Supplement: Supplementary file 1 — Supplementary Figure 1 [file 41416_2018_309_MOESM1_ESM.pptx]

## Slide 1
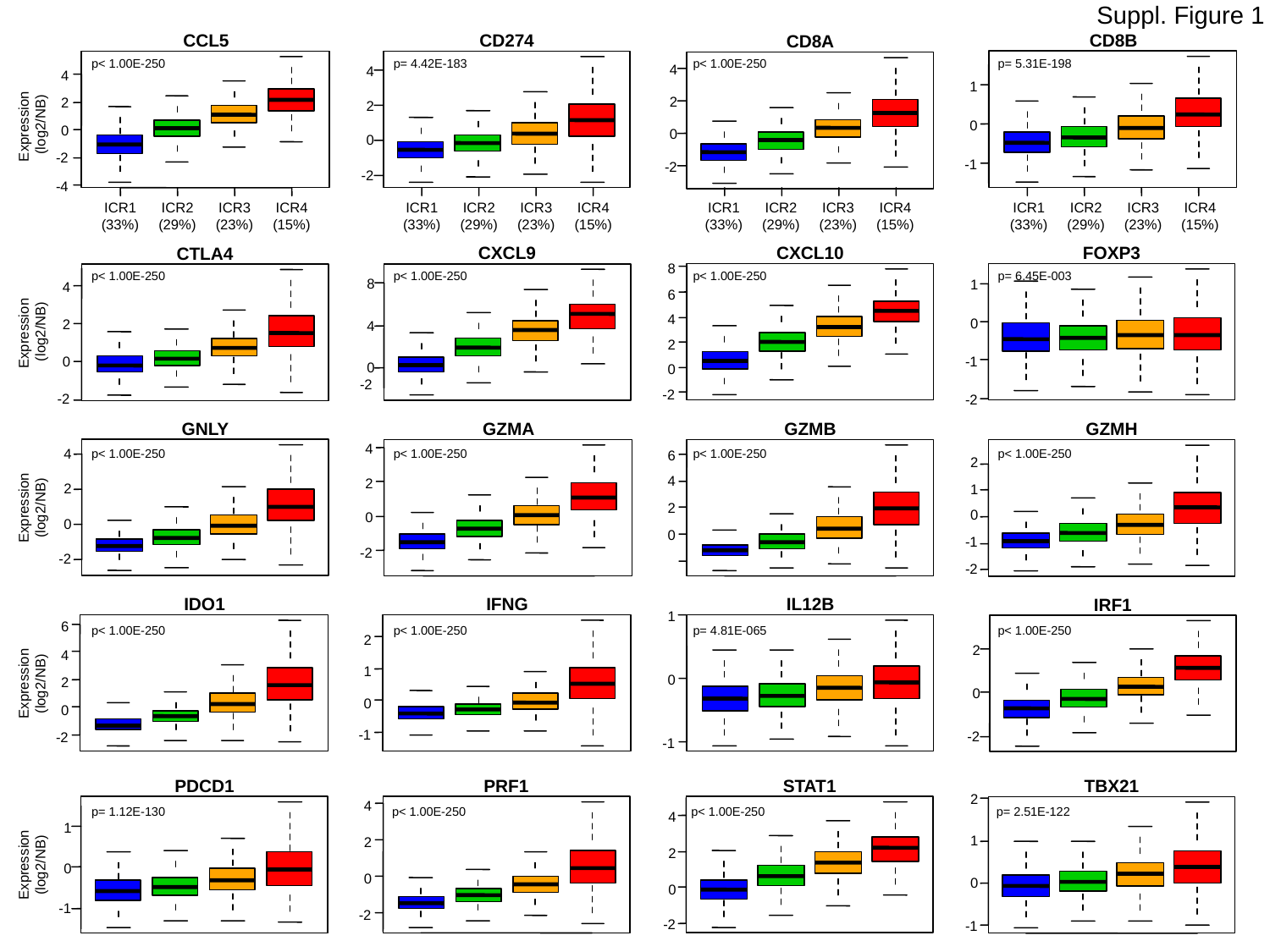

Suppl. Figure 1
CD8B
1
0
-1
CD274
4
2
0
-2
CCL5
4
2
0
-2
-4
CD8A
4
2
0
-2
p< 1.00E-250
p= 4.42E-183
p< 1.00E-250
p= 5.31E-198
p< 1.00E-250
p< 1.00E-250
p< 1.00E-250
p= 6.45E-003
p< 1.00E-250
p< 1.00E-250
p< 1.00E-250
p< 1.00E-250
p< 1.00E-250
p< 1.00E-250
p= 4.81E-065
p< 1.00E-250
p= 1.12E-130
p< 1.00E-250
p< 1.00E-250
p= 2.51E-122
Expression
 (log2/NB)
ICR1
(33%)
ICR2
(29%)
ICR3
(23%)
ICR4
(15%)
ICR1
(33%)
ICR2
(29%)
ICR3
(23%)
ICR4
(15%)
ICR1
(33%)
ICR2
(29%)
ICR3
(23%)
ICR4
(15%)
ICR1
(33%)
ICR2
(29%)
ICR3
(23%)
ICR4
(15%)
FOXP3
1
0
-1
-2
CXCL10
8
6
4
2
0
-2
CXCL9
8
4
0
CTLA4
4
2
0
-2
Expression
 (log2/NB)
-2
GNLY
4
2
0
-2
GZMA
4
2
0
-2
GZMB
6
4
2
0
GZMH
2
1
0
-1
-2
Expression
(log2/NB)
IFNG
2
1
0
-1
IL12B
1
0
-1
IDO1
6
4
2
0
-2
IRF1
2
0
-2
Expression
(log2/NB)
STAT1
4
2
0
-2
PDCD1
1
0
-1
PRF1
4
2
0
-2
TBX21
2
1
0
-1
Expression
(log2/NB)
