# Supplementary material for: The immunologic constant of rejection classification refines the prognostic value of conventional prognostic signatures in breast cancer
Source: Br J Cancer. 2018 Oct 24;119(11):1383–91. doi: 10.1038/s41416-018-0309-1 (PMC6265245; doi:10.1038/s41416-018-0309-1)
Supplement: Supplementary file 2 — Supplementary Figure 2 [file 41416_2018_309_MOESM2_ESM.ppt]

## Slide 1
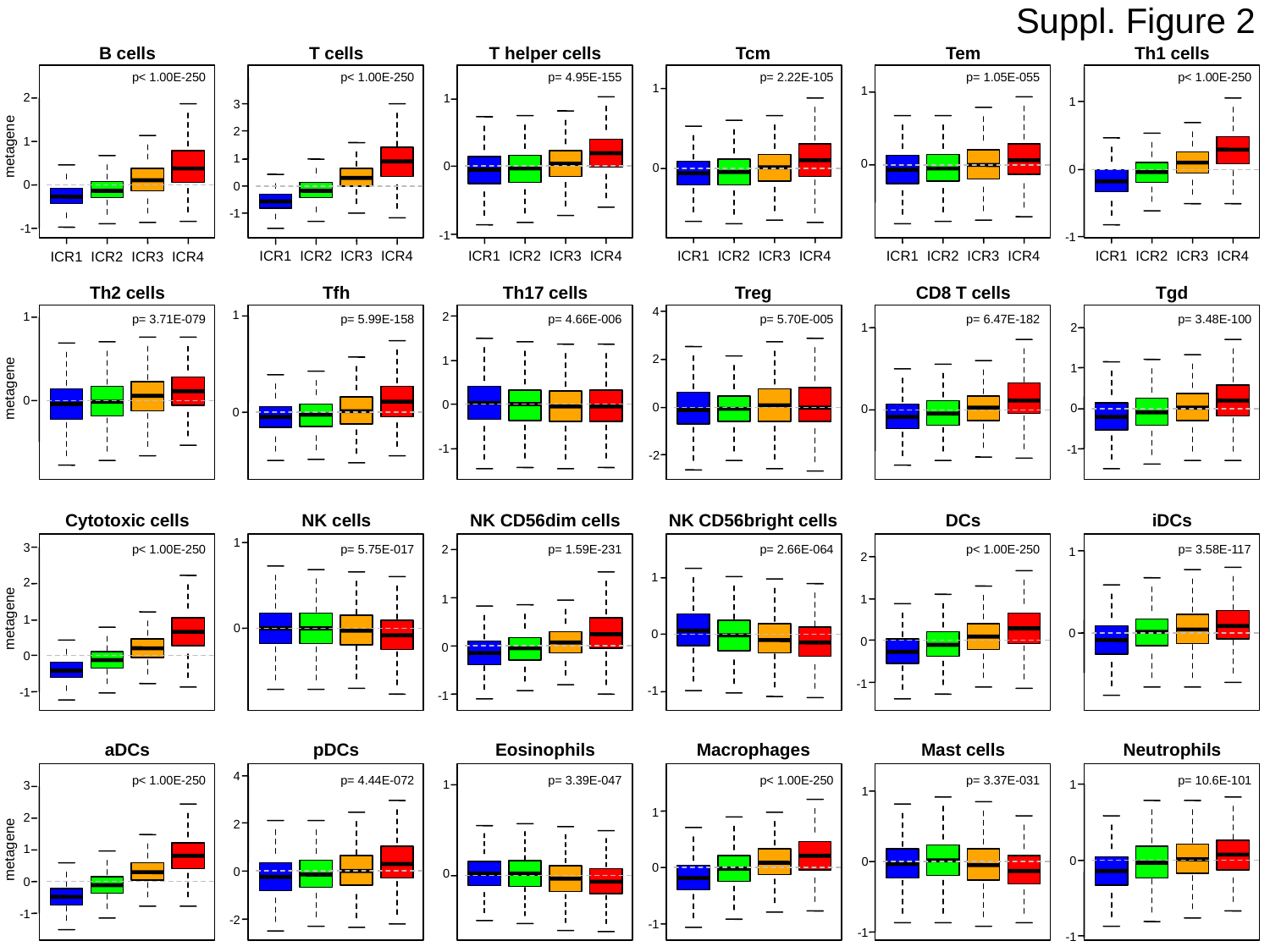

Suppl. Figure 2
B cells
T cells
T helper cells
Tcm
Tem
Th1 cells
p< 1.00E-250
p< 1.00E-250
p= 4.95E-155
p= 2.22E-105
p= 1.05E-055
p< 1.00E-250
1
1
2
1
1
3
2
1
metagene
1
0
0
0
0
0
0
-1
-1
-1
-1
ICR1
ICR2
ICR3
ICR4
ICR1
ICR2
ICR3
ICR4
ICR1
ICR2
ICR3
ICR4
ICR1
ICR2
ICR3
ICR4
ICR1
ICR2
ICR3
ICR4
ICR1
ICR2
ICR3
ICR4
Th2 cells
Tfh
Th17 cells
Treg
CD8 T cells
Tgd
4
1
1
2
p= 3.71E-079
p= 5.99E-158
p= 4.66E-006
p= 5.70E-005
p= 6.47E-182
p= 3.48E-100
1
2
2
1
1
metagene
0
0
0
0
0
0
-1
-1
-2
Cytotoxic cells
NK cells
NK CD56dim cells
NK CD56bright cells
DCs
iDCs
1
3
p< 1.00E-250
p= 5.75E-017
p= 1.59E-231
p= 2.66E-064
p< 1.00E-250
p= 3.58E-117
2
1
2
1
2
1
1
metagene
1
0
0
0
0
0
0
-1
-1
-1
-1
aDCs
pDCs
Eosinophils
Macrophages
Mast cells
Neutrophils
4
p< 1.00E-250
p= 4.44E-072
p= 3.39E-047
p< 1.00E-250
p= 3.37E-031
p= 10.6E-101
1
1
3
1
1
2
2
1
metagene
0
0
0
0
0
0
-1
-2
-1
-1
-1
