# Supplementary material for: The immunologic constant of rejection classification refines the prognostic value of conventional prognostic signatures in breast cancer
Source: Br J Cancer. 2018 Oct 24;119(11):1383–91. doi: 10.1038/s41416-018-0309-1 (PMC6265245; doi:10.1038/s41416-018-0309-1)
Supplement: Supplementary file 3 — Supplementary Figure 3 [file 41416_2018_309_MOESM3_ESM.pptx]

## Slide 1
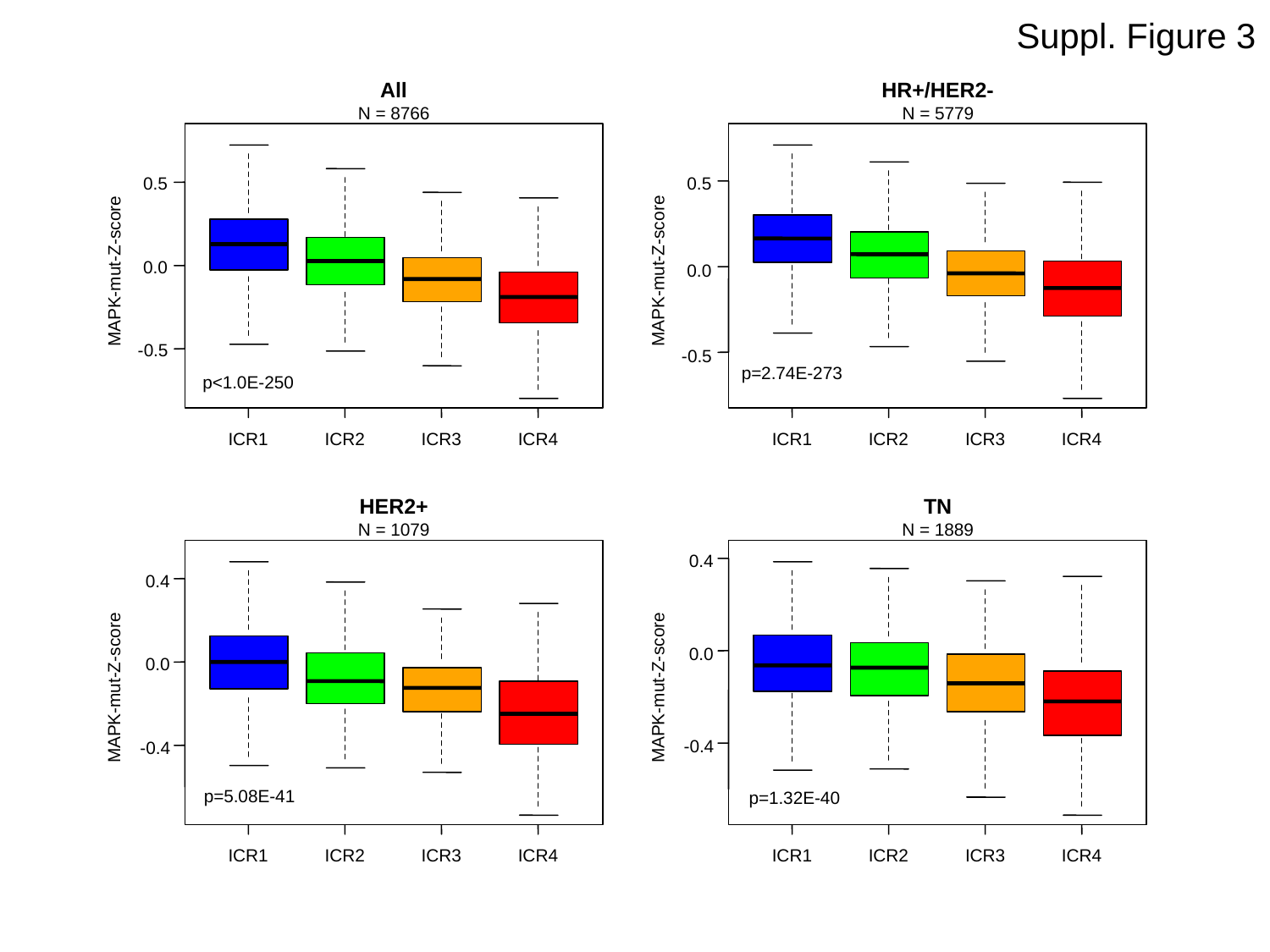

Suppl. Figure 3
All
N = 8766
HR+/HER2-
N = 5779
0.5
0.5
0.0
0.0
MAPK-mut-Z-score
MAPK-mut-Z-score
-0.5
-0.5
p=2.74E-273
p<1.0E-250
ICR1
ICR2
ICR3
ICR4
ICR1
ICR2
ICR3
ICR4
HER2+
N = 1079
0.4
0.4
0.0
0.0
MAPK-mut-Z-score
-0.4
-0.4
p=5.08E-41
ICR1
ICR2
ICR3
ICR4
ICR1
ICR2
ICR3
ICR4
TN
N = 1889
MAPK-mut-Z-score
p=1.32E-40
