# Supplementary material for: The immunologic constant of rejection classification refines the prognostic value of conventional prognostic signatures in breast cancer
Source: Br J Cancer. 2018 Oct 24;119(11):1383–91. doi: 10.1038/s41416-018-0309-1 (PMC6265245; doi:10.1038/s41416-018-0309-1)
Supplement: Supplementary file 4 — Supplementary Figure 4 [file 41416_2018_309_MOESM4_ESM.pptx]

## Slide 1
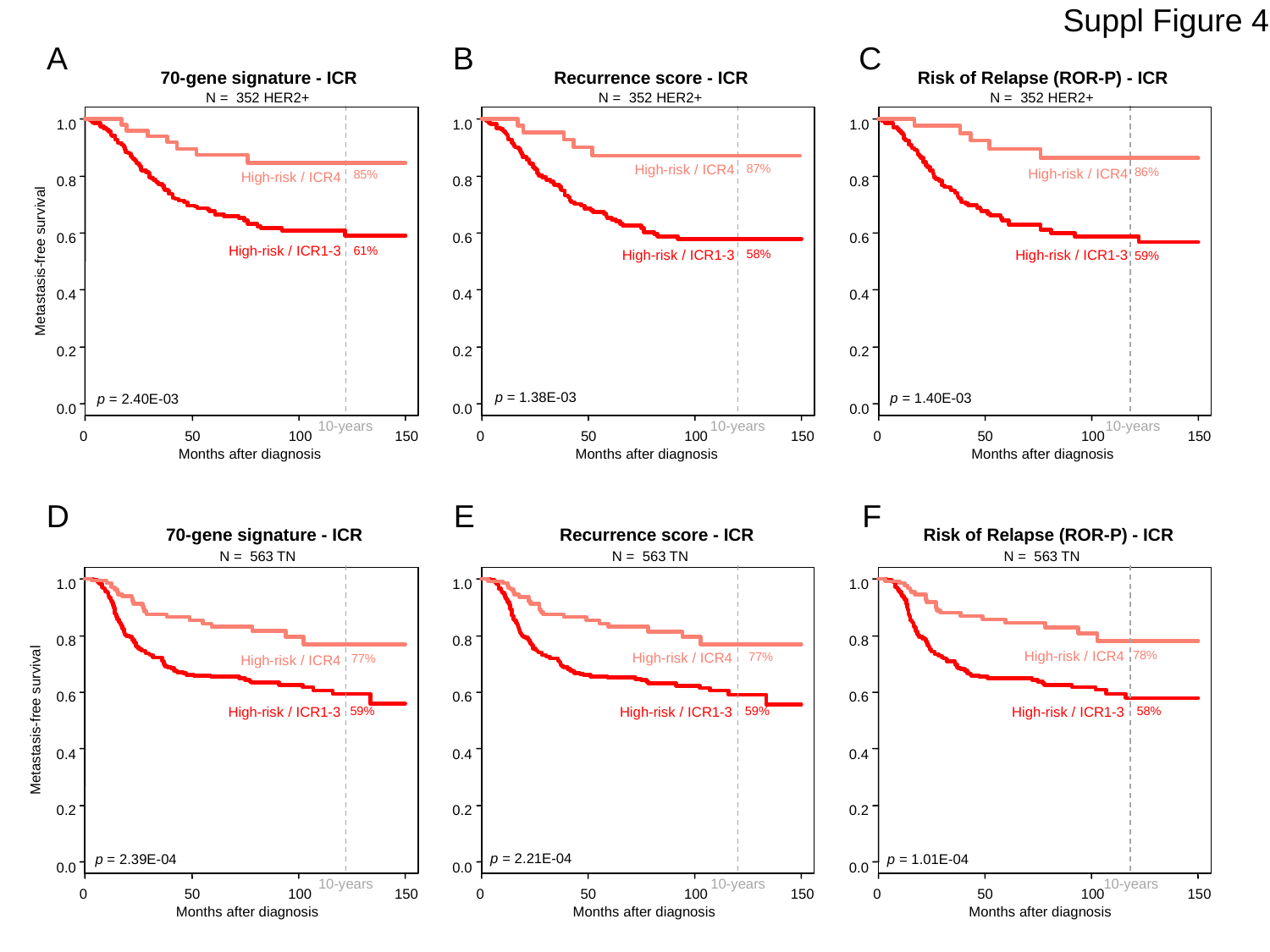

Suppl Figure 4
B
C
A
70-gene signature - ICR
Recurrence score - ICR
Risk of Relapse (ROR-P) - ICR
N = 352 HER2+
N = 352 HER2+
N = 352 HER2+
87%
86%
85%
61%
58%
59%
10-years
10-years
10-years
E
F
D
70-gene signature - ICR
Recurrence score - ICR
Risk of Relapse (ROR-P) - ICR
N = 563 TN
N = 563 TN
N = 563 TN
78%
77%
77%
58%
59%
59%
10-years
10-years
10-years
1.0
1.0
1.0
0.8
0.8
0.8
0.6
0.6
0.6
Metastasis-free survival
0.4
0.4
0.4
0.2
0.2
0.2
p = 1.38E-03
p = 1.40E-03
p = 2.40E-03
0.0
0.0
0.0
0
50
100
150
0
50
100
150
0
50
100
150
Months after diagnosis
Months after diagnosis
Months after diagnosis
High-risk / ICR4
High-risk / ICR4
High-risk / ICR4
High-risk / ICR1-3
High-risk / ICR1-3
High-risk / ICR1-3
1.0
1.0
1.0
0.8
0.8
0.8
0.6
0.6
0.6
Metastasis-free survival
0.4
0.4
0.4
0.2
0.2
0.2
p = 2.21E-04
p = 2.39E-04
p = 1.01E-04
0.0
0.0
0.0
0
50
100
150
0
50
100
150
0
50
100
150
Months after diagnosis
Months after diagnosis
Months after diagnosis
High-risk / ICR4
High-risk / ICR4
High-risk / ICR4
High-risk / ICR1-3
High-risk / ICR1-3
High-risk / ICR1-3
