# Supplementary material for: The immunologic constant of rejection classification refines the prognostic value of conventional prognostic signatures in breast cancer
Source: Br J Cancer. 2018 Oct 24;119(11):1383–91. doi: 10.1038/s41416-018-0309-1 (PMC6265245; doi:10.1038/s41416-018-0309-1)
Supplement: Supplementary file 5 — Supplementary Figure 5 [file 41416_2018_309_MOESM5_ESM.pptx]

## Slide 1
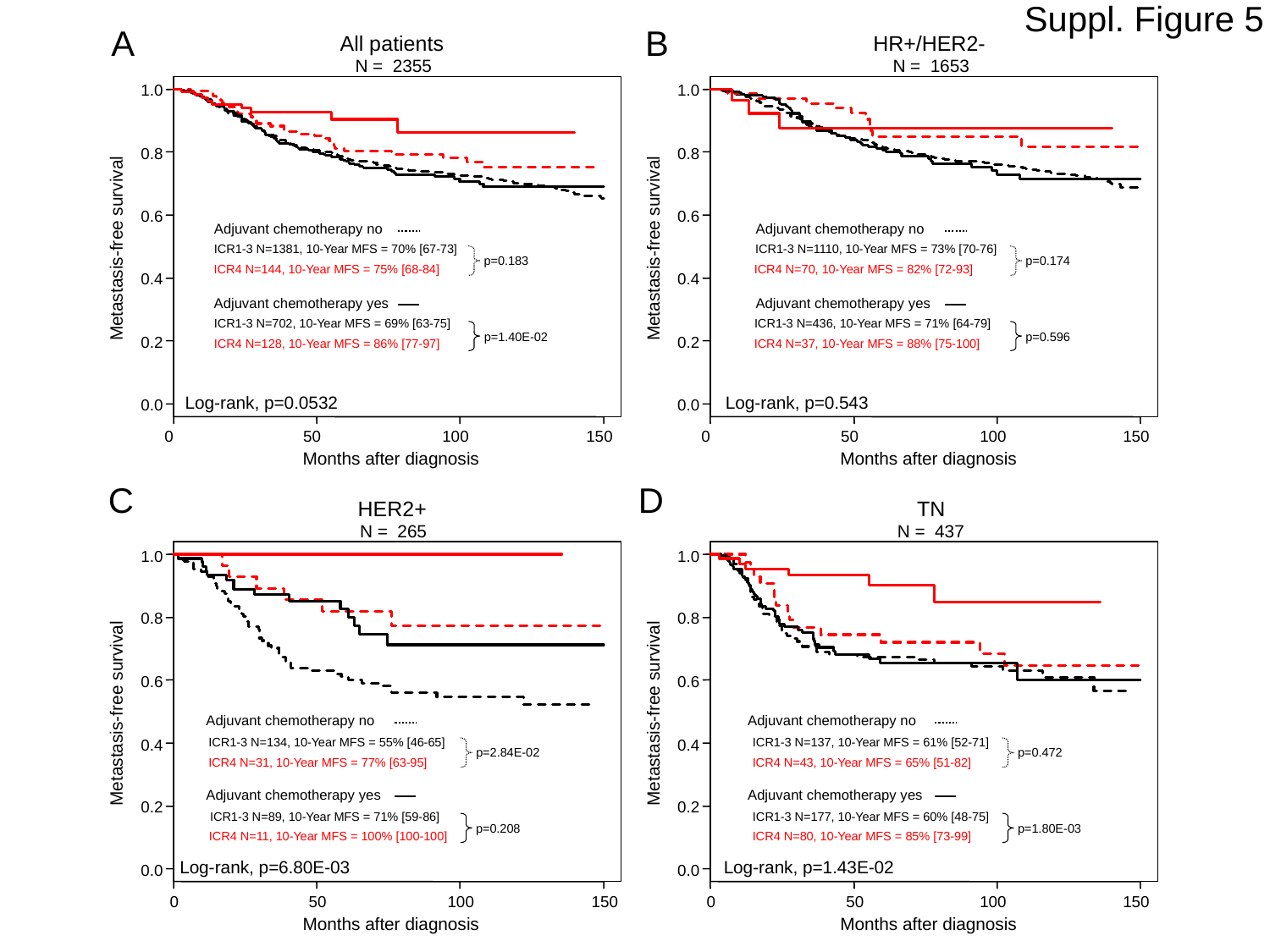

Suppl. Figure 5
A
B
All patients
HR+/HER2-
N = 2355
N = 1653
1.0
1.0
0.8
0.8
0.6
0.6
Metastasis-free survival
Metastasis-free survival
ICR1-3 N=1381, 10-Year MFS = 70% [67-73]
ICR1-3 N=1110, 10-Year MFS = 73% [70-76]
ICR4 N=144, 10-Year MFS = 75% [68-84]
ICR4 N=70, 10-Year MFS = 82% [72-93]
0.4
0.4
ICR1-3 N=702, 10-Year MFS = 69% [63-75]
ICR1-3 N=436, 10-Year MFS = 71% [64-79]
0.2
0.2
ICR4 N=128, 10-Year MFS = 86% [77-97]
ICR4 N=37, 10-Year MFS = 88% [75-100]
Log-rank, p=0.0532
Log-rank, p=0.543
0.0
0.0
0
50
100
150
0
50
100
150
Months after diagnosis
Months after diagnosis
HER2+
TN
N = 265
N = 437
1.0
1.0
0.8
0.8
0.6
0.6
Metastasis-free survival
Metastasis-free survival
0.4
ICR1-3 N=134, 10-Year MFS = 55% [46-65]
0.4
ICR1-3 N=137, 10-Year MFS = 61% [52-71]
ICR4 N=31, 10-Year MFS = 77% [63-95]
ICR4 N=43, 10-Year MFS = 65% [51-82]
0.2
0.2
ICR1-3 N=89, 10-Year MFS = 71% [59-86]
ICR1-3 N=177, 10-Year MFS = 60% [48-75]
ICR4 N=11, 10-Year MFS = 100% [100-100]
ICR4 N=80, 10-Year MFS = 85% [73-99]
Log-rank, p=6.80E-03
Log-rank, p=1.43E-02
0.0
0.0
0
50
100
150
0
50
100
150
Months after diagnosis
Months after diagnosis
Adjuvant chemotherapy no
Adjuvant chemotherapy yes
p=0.183
p=1.40E-02
Adjuvant chemotherapy no
Adjuvant chemotherapy yes
p=0.174
p=0.596
C
D
Adjuvant chemotherapy no
Adjuvant chemotherapy yes
p=2.84E-02
p=0.208
Adjuvant chemotherapy no
Adjuvant chemotherapy yes
p=0.472
p=1.80E-03
